# Management of Single Tooth Replacement in the Esthetic Region Using Immediate Implant Placement: A Case Report

**DOI:** 10.1002/ccr3.70089

**Published:** 2025-01-06

**Authors:** Manish Rathi, Balaji Manohar

**Affiliations:** ^1^ Department of Periodontology, Kalinga Institute of Dental Sciences Kalinga Institute of Industrial Technology (Deemed to Be University) Bhubaneswar Odisha India; ^2^ Department of Periodontology Army Dental Centre Research and Referral Delhi India

**Keywords:** bone, dental esthetic, dental implant loading, immediate, regeneration

## Abstract

This case report aimed to illustrate the management of single tooth replacement in the esthetic region using immediate implant placement, highlighting the importance of interdisciplinary treatment planning and adherence to esthetic principles. A 37‐year‐old female presented with a fractured upper front tooth, necessitating immediate attention to restore esthetic.

**Summary**

- Immediate implant is predictable method for placing implant in anterior esthetic region. Its key advantages include shorter treatment time, reduced surgical morbidity.
- This case presentation is unique as it provides a step‐by‐step detailed management using SAC Assessment Tool by International Team of Implantologists (ITI) and therefore complication management to correct the esthetics by second‐stage follow‐up procedure.
- Correction of “microesthetics” was also carried out, thereby improving “tangible benefits” to the patients for which patient was unaware, including 3‐year postoperative review.

## 1 Introduction

Achieving a lovely smile involves considerations beyond mere functionality, encompassing both functional and esthetic aspects [1]. Integrated treatment planning is frequently required to successfully reconcile face characteristics and dental proportions [2]. Orthodontic interventions may precede esthetic rehabilitation to minimize invasive procedures [3]. Soft tissue management plays a crucial role in achieving integration between soft and hard tissues [4, 5], while digital resources enable virtual patient simulations for comprehensive treatment planning. Immediate implant in esthetic region with immediate implant provisionalization (IIP) have been proven to be successful with minimal requirement of hard and soft tissue augmentation and maximum preservation of the soft tissue architecture [6]. Significant advancements in bone graft substitutes to be used for hard tissue augmentation have also been reported [7]. Similarly, when a thin phenotype is present, simultaneous soft tissue augmentation using connective tissue graft (CTG) can be considered to improve the stability of overall peri‐implant mucosal margin as well as the underlying bone [8].

With technological advancements, various non‐invasive methods have been developed overtime to assess the soft tissue thickness. 3D‐digital analysis using the digital scanner for analysis of soft tissue thickness and volume have been reported in the literature [9]. Apart from this, successful integration of implant requires good primary stability which can be measured using Insertion torque as well as resonance frequency analysis (RFA) and denoted in terms of implant stability quotient (ISQ) values [10]. Implant‐abutment connection with morse taper design or conical connection has little micromovements, resulting in better marginal bone stability. Gehrke et al. in their study showed stable marginal bone levels where longer titanium base have been used in conical implants [11]. Implants with smaller conical connection have more frictional resistance and thus reduces the detorque of abutment screw [12]. Other factors affecting success of dental implants include implant macrogeometry and implant surface topography [13].

Immediate implants placement in the infected region has also been a controversial subject. However, recent guidelines suggest that infection is not a contraindication for Implant placement. This case report explores the utilization of IIP for single tooth replacement in the esthetic zone, emphasizing optimal esthetic outcomes with high predictability and minimal risk of complications [14].

## 2 Case Presentation

**FIGURE 1:**
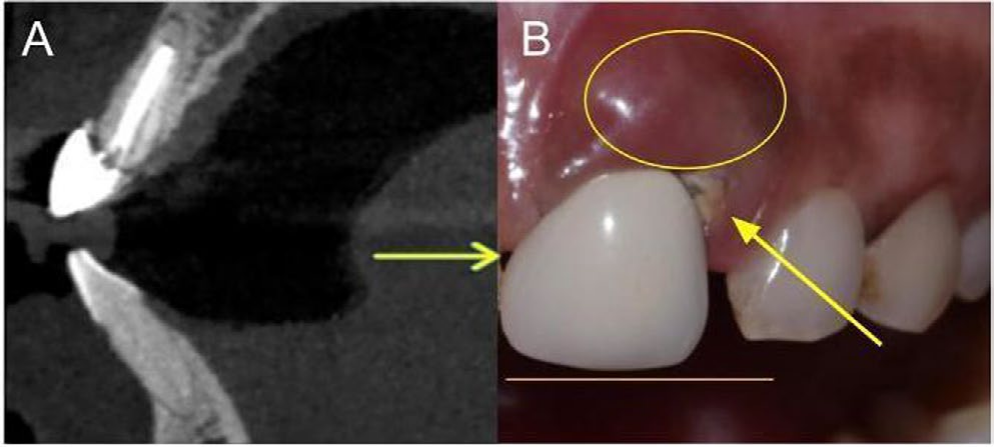
Initial situation. (A) Cone beam computed tomography. (B) Fractured upper front tooth, with evidence of swelling for a duration of 3 days (yellow circle). Evidence of deterioration is shown with arrows.

A consenting 37‐year‐old female patient presented with a chief complaint of a fractured upper front tooth accompanied by swelling for 3 days (see Figure 1). Past dental history revealed root canal treatment 1 year prior, followed by restoration with a porcelain‐fused‐to‐metal (PFM) crown. General medical, dental, and family histories were unremarkable. Clinical examination indicated a well‐oriented patient with moderate build and adequate nourishment. Vital signs were within normal limits. Preoperative assessment was carried out using the SAC assessment tool of ITI. The surgical procedure is “Complex.” SAC surgical assessment also revealed that there was a high esthetic complication associated with the procedure and suggested simultaneous bone augmentation along with adjunctive soft tissue grafting, so the case was planned considering the ITI treatment guidelines.

## 3 Methods

**FIGURE 2:**
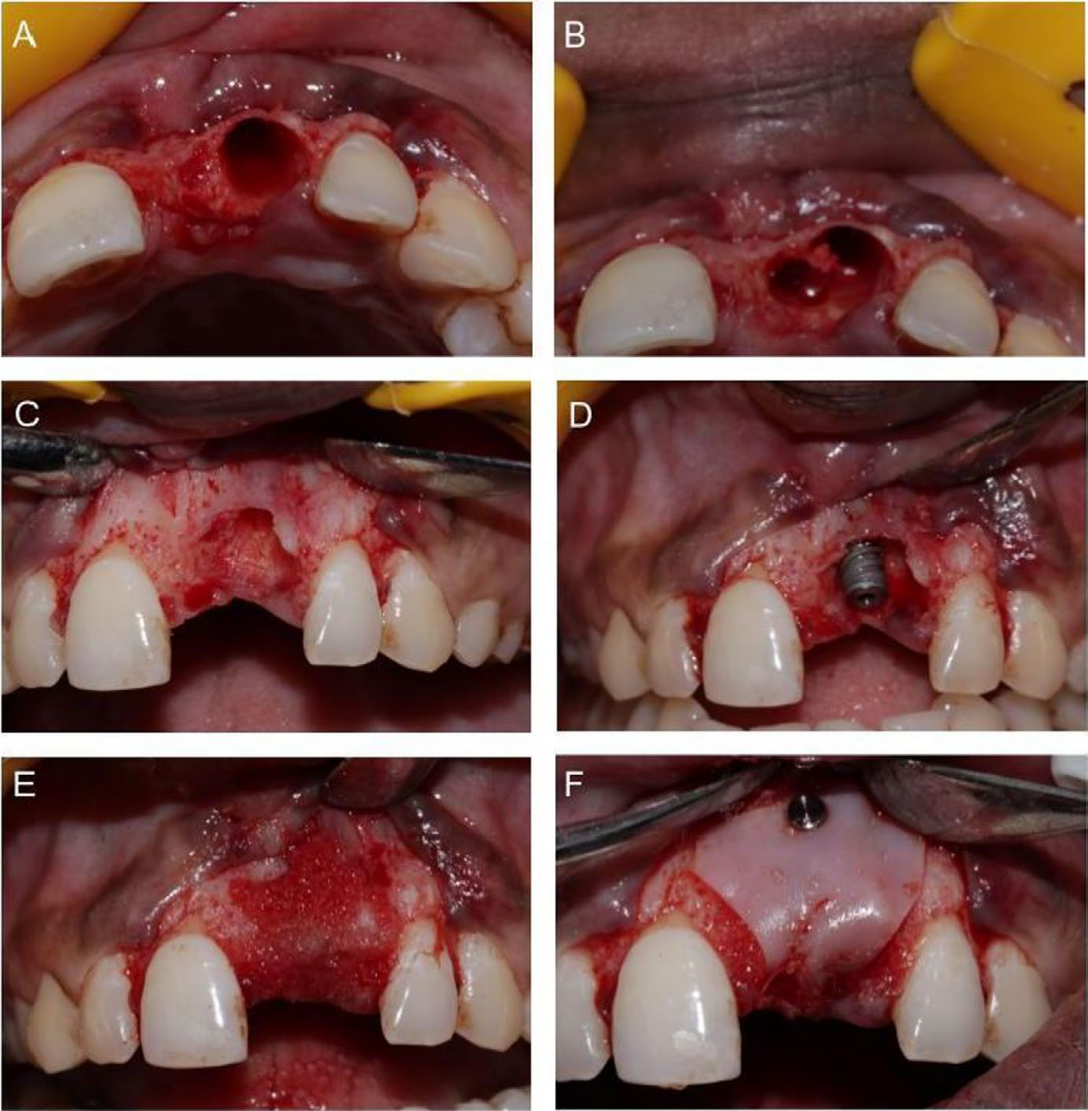
(A) Extraction socket with respect to 21. (B) Implant site prep. (C) Bony defect post‐atraumatic extraction wrt 21. (D) Immediate implant placed. (E) GBR using alloplast bone graft. (F) Collagen membrane stabilized using tac pin.

**FIGURE 3:**
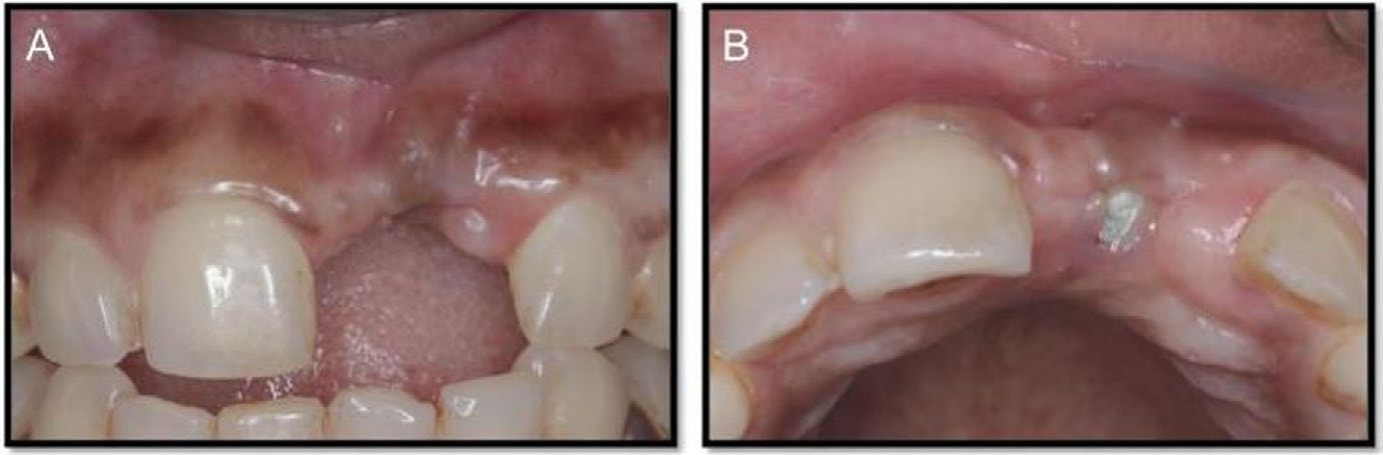
Postoperative. (A) Anterior view. (B) Occlusal view showing labial deficiency in volume compared with adjacent tooth.

The surgical approach involved mucoperiosteal flap elevation to access the bony defect post‐atraumatic extraction (Figure 2). Immediate implant placement was performed, followed by guided bone regeneration (GBR) using alloplast bone graft with collagen membrane stabilization. Subsequent closure of the surgical site ensured optimal healing conditions (Figure 3). After 06 months, the surgical site revealed satisfactory hard and soft tissue ridge dimensions in apico‐coronal direction. However, there was soft tissue ridge deficiency in the buccolingual direction when viewed from the occlusal aspect. Therefore, second‐stage soft tissue grafting procedure using CTG was planned.

**FIGURE 4:**
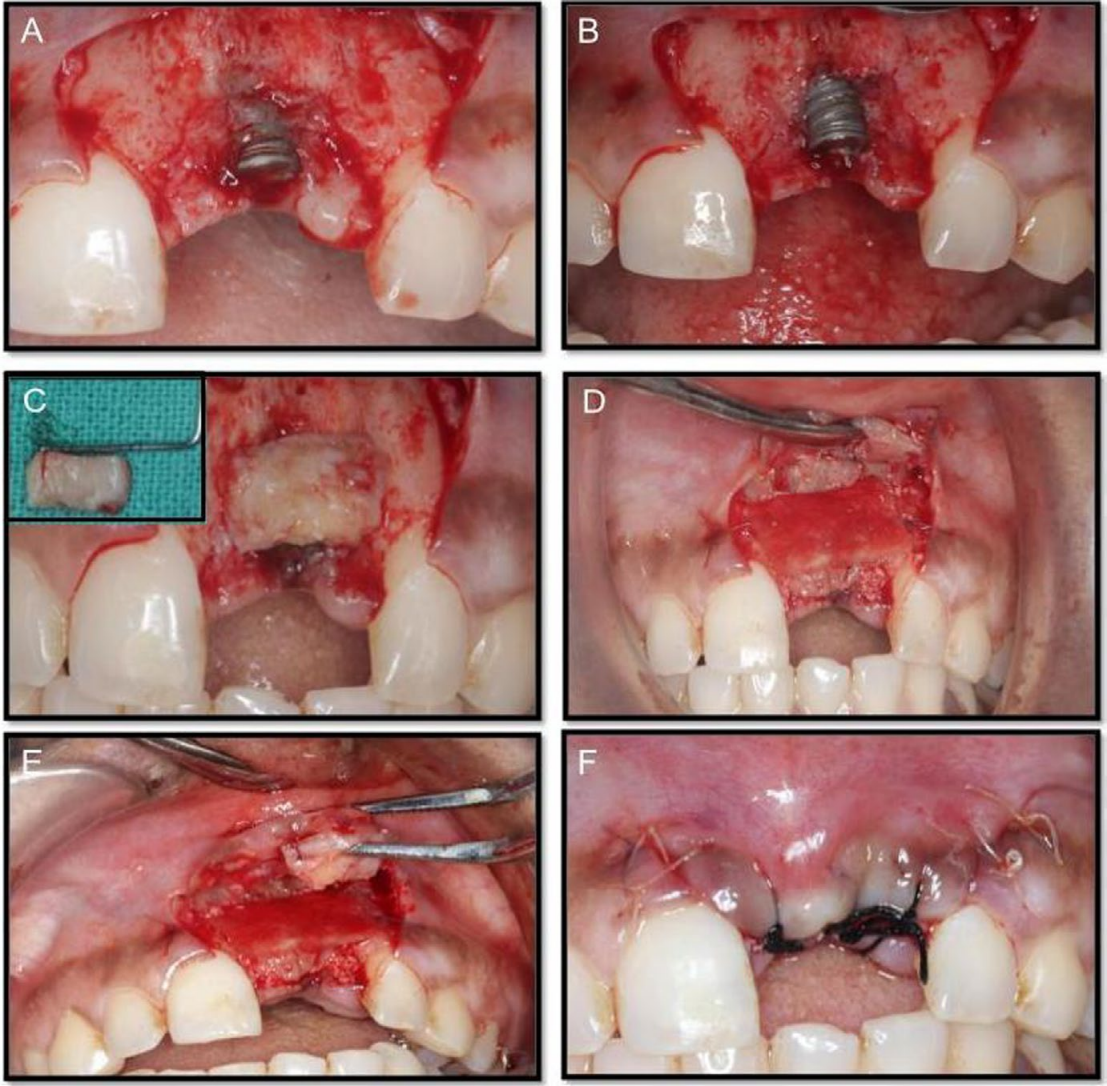
(A, B) Implant exposure. (C) Inset: connective tissue graft (CTG) harvested from palate. Graft checked for the dimensions. (D) Guided bone regeneration using mixture of autologous and xenograft with collagen membrane. (E) CTG sutured on the inner aspect of the labial flap. (F) Primary closure of flap.

**FIGURE 5:**
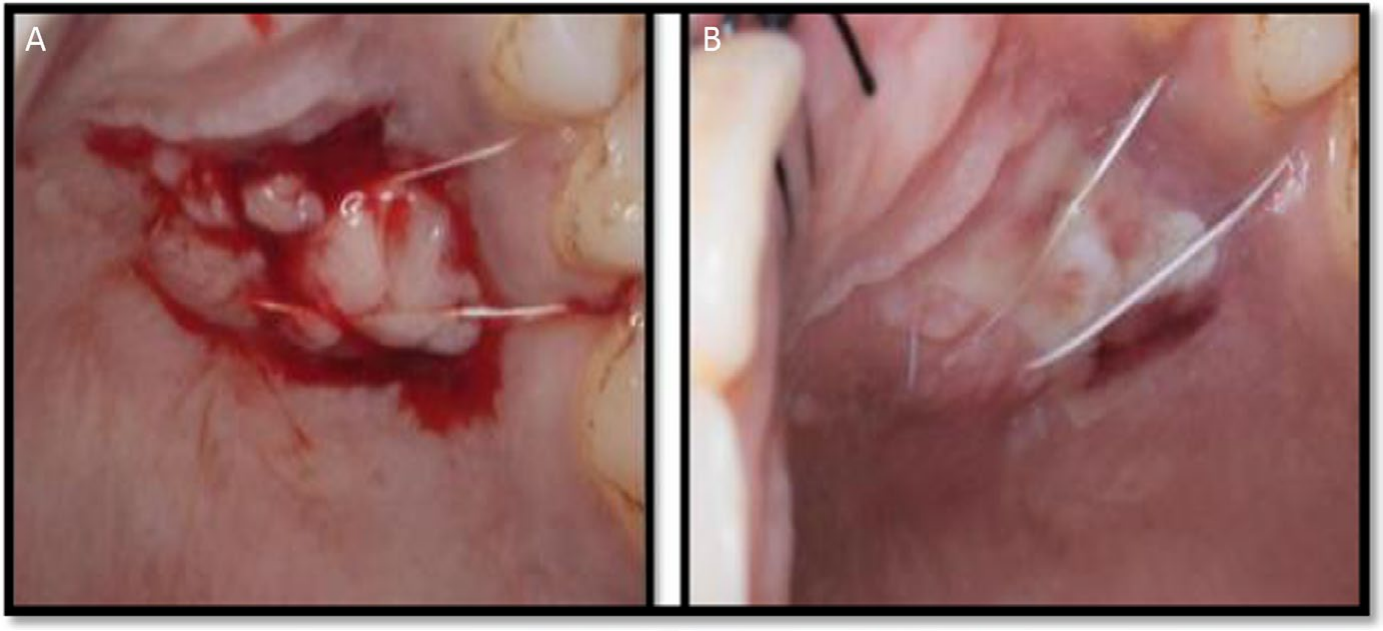
(A) Platelet‐rich fibrin as palatal bandage. (B) Palatal wound healing after 48 h.

**FIGURE 6:**
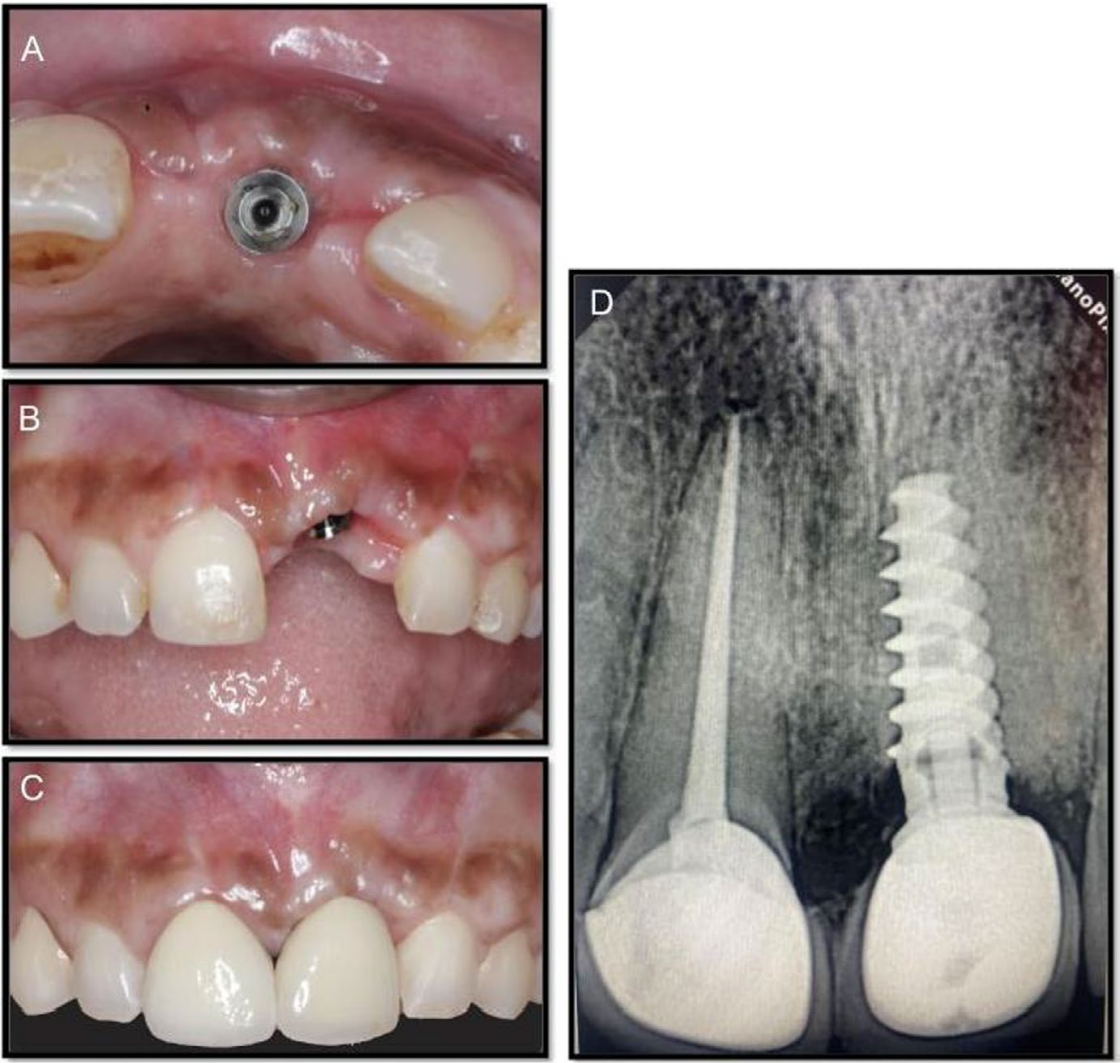
Postoperative (6 months). (A) Occlusal view of implant. (B) Frontal view of implant. (C) Anterior view of full implant. (D) Radiovisiograph of implant.

After full thickness mucoperiosteal elevation was carried out, implant thread exposure was evident which was treated with GBR procedure using a mixture of autogenous graft and xenograft. Particulate autogenous graft was harvested from the operative site apical to the implant using 4.0 mm diameter trephine burs. The surgical site is then covered with a resorbable collagen membrane, which is stabilized and secured with 5‐0 vicryl sutures. During the second‐stage surgical procedure, a CTG harvested from the palate was sutured onto the inner aspect of the labial flap to enhance soft tissue volume and contour (Figure 4). Platelet‐rich fibrin (PRF) application served as a palatal bandage to facilitate wound healing (Figures 5 and 6).

## 4 Results

At the 6‐month postoperative evaluation, the implant demonstrated primary stability with an insertion torque of 50Ncm. Pink esthetic score (PES) and white esthetic score (WES) assessments revealed significant improvements compared with preoperative values, indicating favorable esthetic outcomes. Microesthetics were corrected by restoring 11 with ceramic crown and esthetic restoration on 22.

## 5 Discussion

In this study, we aimed to address several hypotheses related to the effectiveness of IIP in achieving optimal esthetic outcomes in the anterior maxilla. Our first hypothesis was that IIP would result in favorable esthetic outcomes with high predictability and minimal risk of complications. The experimental data upheld this hypothesis, as demonstrated by the significant improvements in the PES and WES assessments at the 6‐month postoperative evaluation (Table 1). These findings are consistent with previous research by Chen and Buser (2014) [14], who reported favorable esthetic outcomes following IIP in the anterior maxilla.

**TABLE 1:** Pink esthetic score (PES) and white esthetic score (WES) assessments.

	Preoperative	Postoperative
Pink esthetic score	3	9
White esthetic score	2	9
Total	5/20	18/20

Contrary to our second hypothesis, which anticipated challenges in achieving optimal peri‐implant soft tissue seal, we found that rigorous surgical technique and postoperative care resulted in primary stability of the implant and significant improvements in esthetic outcomes. This contrasts with the concerns raised by Chappuis et al. (2018) [4] regarding the effectiveness of contour augmentation with GBR, suggesting a higher risk of esthetic complications associated with complex surgical procedures. However, in a recent systematic review by Martin et al. (2024), it was concluded that IIP with immediate provisionalization can be considered along with use of biologics and bone grafts for a favorable esthetic outcome [15].

The integration of CTG and PRF application during the second‐stage surgical procedure further contributed to enhanced soft tissue volume and contour, supporting our third hypothesis. These findings are consistent with the recommendations by Castro et al. (2023) suggesting the successful use of regenerative approach for peri‐implant bone augmentation [16], and by Furhauser et al. (2005) [5], who emphasized the importance of soft tissue evaluation in implant dentistry and proposed the PES as a reliable assessment tool. Rondone et al. (2024) recommended the use of bone graft and substitutes along with soft tissue grafts along with immediate implants for better bone and soft tissue stability post placement [17]. Use of resorbable collagen membrane for GBR was placed as primary closure was intended to reduce the enzymatic degradation of collagen, and lesser complications compared with non‐resorbable collagen membranes [18].

**FIGURE 7:**
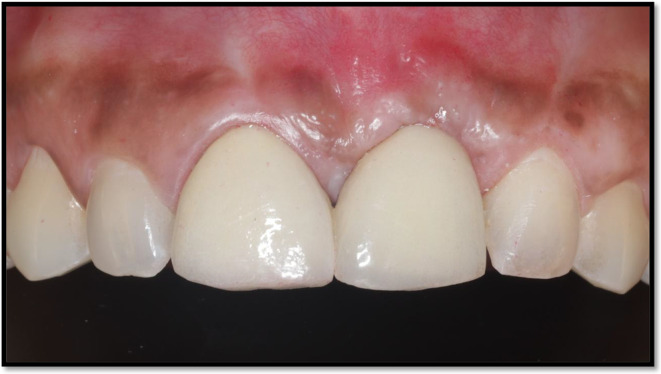
Postoperative (3 years). Stabilized soft tissues with a band of keratinized tissue from the gingival margin.

Combining the knowledge gained from these findings, we conclude that IIP, when performed with rigorous planning and execution, can lead to predictable and favorable esthetic outcomes in the anterior maxilla. This study contributes to the existing literature by providing evidence‐based insights into the management of single tooth replacement in the esthetic zone using IIP. This is further supported by postoperative stable results when patient was reassessed after 3 years (Figure 7).

However, it is essential to acknowledge the limitations of this study. As this is a case report with a 3‐year follow‐up period, this restricts the generalizability of the findings. Furthermore, the study focused primarily on esthetic outcomes and did not assess long‐term implant success rates or patient‐reported outcomes. Future research should aim to address these limitations by conducting larger‐scale prospective studies with longer follow‐up periods, incorporating patient‐reported outcomes and assessing implant survival rates.

## 6 Conclusion

Immediate implant placement necessitates rigorous planning and execution to attain desirable esthetic outcomes. Understanding of hard and soft tissue anatomy, microesthetics, and patient expectations is imperative for evidence‐based treatment planning and successful implant therapy. Integrating interdisciplinary approaches enhances predictability and ensures comprehensive care in restorative dentistry.

## Author Contributions

**Manish Rathi:** conceptualization, data curation, funding acquisition, investigation, methodology, project administration, resources, software, supervision, validation, visualization, writing – original draft, writing – review and editing. **Balaji Manohar:** formal analysis, funding acquisition, supervision, validation, visualization, writing – review and editing.

## Disclosure

The authors declares that they do not have any financial interest in the contents included in this article.

## Ethics Statement

Written informed consent was obtained from the patient for publication of this case report.

## Conflicts of Interest

The authors declare no conflicts of interest.

## Data Availability

The data are available from TBA.
